# Comparison of Single-Breed and Multi-Breed Training Populations for Infrared Predictions of Novel Phenotypes in Holstein Cows

**DOI:** 10.3390/ani11071993

**Published:** 2021-07-02

**Authors:** Lucio Flavio Macedo Mota, Sara Pegolo, Toshimi Baba, Gota Morota, Francisco Peñagaricano, Giovanni Bittante, Alessio Cecchinato

**Affiliations:** 1 Department of Agronomy, Food, Natural Resources, Animals and Environment (DAFNAE), University of Padova, 35020 Legnaro, Italy; flaviommota.zoo@gmail.com (L.F.M.M.); sara.pegolo@unipd.it (S.P.); giovanni.bittante@unipd.it (G.B.); 2 Department of Animal and Poultry Sciences, Virginia Polytechnic Institute and State University, Blacksburg, VA 24061, USA; tbaba@vt.edu (T.B.); morota@vt.edu (G.M.); 3 Center for Advanced Innovation in Agriculture, Virginia Polytechnic Institute and State University, Blacksburg, VA 24061, USA; 4 Department of Animal and Dairy Sciences, University of Wisconsin-Madison, Madison, WI 53706, USA; fpenagarican@wisc.edu

**Keywords:** dual-purpose dairy breed, Fourier-transform infrared, specialized dairy breed, validation strategies

## Abstract

**Simple Summary:**

In order to obtain accurate infrared predictions, a large number of training animals are needed, aiming to increase the predictive ability of Fourier-transform infrared (FTIR) predictions. In this study, we compared different validation scenarios that involved combining specialized and dual-purpose dairy breeds in the training population FTIR predictions for three different phenotypes in the major cattle breed, i.e., Holstein cattle. Results show that the design of the training population is an important factor in improving predictive ability in the Holstein breed with potential implications also for the minor breeds. However, this improvement is limited by the phenotypic variability of traits of concern and spectral variability between the training and validation sets and the number of animals in the training population.

**Abstract:**

In general, Fourier-transform infrared (FTIR) predictions are developed using a single-breed population split into a training and a validation set. However, using populations formed of different breeds is an attractive way to design cross-validation scenarios aimed at increasing prediction for difficult-to-measure traits in the dairy industry. This study aimed to evaluate the potential of FTIR prediction using training set combining specialized and dual-purpose dairy breeds to predict different phenotypes divergent in terms of biological meaning, variability, and heritability, such as body condition score (BCS), serum β-hydroxybutyrate (BHB), and kappa casein (k-CN) in the major cattle breed, i.e., Holstein-Friesian. Data were obtained from specialized dairy breeds: Holstein (468 cows) and Brown Swiss (657 cows), and dual-purpose breeds: Simmental (157 cows), Alpine Grey (75 cows), and Rendena (104 cows), giving a total of 1461 cows from 41 multi-breed dairy herds. The FTIR prediction model was developed using a gradient boosting machine (GBM), and predictive ability for the target phenotype in Holstein cows was assessed using different cross-validation (CV) strategies: a within-breed scenario using 10-fold cross-validation, for which the Holstein population was randomly split into 10 folds, one for validation and the remaining nine for training (10-fold_HO); an across-breed scenario (BS_HO) where the Brown Swiss cows were used as the training set and the Holstein cows as the validation set; a specialized multi-breed scenario (BS+HO_10-fold), where the entire Brown Swiss and Holstein populations were combined then split into 10 folds, and a multi-breed scenario (Multi-breed), where the training set comprised specialized (Holstein and Brown Swiss) and dual-purpose (Simmental, Alpine Grey, and Rendena) dairy cows, combined with nine folds of the Holstein cows. Lastly a Multi-breed CV2 scenario was implemented, assuming the same number of records as the reference scenario and using the same proportions as the multi-breed. Within-Holstein, FTIR predictions had a predictive ability of 0.63 for BCS, 0.81 for BHB, and 0.80 for k-CN. Using a specific breed (Brown Swiss) as the training set for prediction in the Holstein population reduced the prediction accuracy by 10% for BCS, 7% for BHB, and 11% for k-CN. Notably, the combination of Holstein and Brown Swiss cows in the training set increased the predictive ability of the model by 6%, which was 0.66 for BCS, 0.85 for BHB, and 0.87 for k-CN. Using multiple specialized and dual-purpose animals in the training set outperforms the 10-fold_HO (standard) approach, with an increase in predictive ability of 8% for BCS, 7% for BHB, and 10% for k-CN. When the Multi-breed CV2 was implemented, no improvement was observed. Our findings suggest that FTIR prediction of different phenotypes in the Holstein breed can be improved by including different specialized and dual-purpose breeds in the training population. Our study also shows that predictive ability is enhanced when the size of the training population and the phenotypic variability are increased.

## 1. Introduction

Fourier-transform infrared spectroscopy (FTIR) technique is used to obtain the infrared spectra of absorption, emission, and photoconductivity of solids, liquids, and gases. It measures the vibration and rotation of molecules determined by infrared radiation at a specific wavelength [1]. In the animal breeding context, the increasing availability of genomic information has pushed the practice towards the implementation of high-throughput phenotyping techniques such as FTIR, which is able to generate real-time, non-invasive, accurate phenotypic predictions at the population level. In dairy cattle, FTIR spectroscopy is extensively applied to the milk matrix for the prediction of standard milk composition, but it has been also proven to be a useful tool for predicting phenotypes that are difficult or expensive to measure such as milk fatty acids [2], milk proteins [3], methane emission [4], fat, and animals’ metabolic and production efficiency [5]. In the context of genomic selection, the use of a multi-breed framework was investigated with the purpose to increase prediction accuracy for difficult to measure traits in dairy cattle [6].

Phenotypic prediction using FTIR milk spectra requires the construction of calibration and validation sets, generally from a small dataset, which may affect the predictive ability, especially for complex phenotypes. A solution to this problem might be to combine information from different breeds and/or populations in the training set. Indeed, some studies in the field of genomic selection have done so, and this has increased the available information for developing the calibration equations, resulting in improved model predictive ability and robustness [7,8,9,10]. However, in FTIR prediction, different cross-validation (CV) strategies have not yet been evaluated. In particular, integrating information from different breeds to increase sample size and phenotypic variability could be useful to improve predictive ability [7].

Using multi-breed information in a training dataset is an attractive tool to numerically increase the training set and obtain accurate predictions. A critical step towards implementing FTIR prediction using different breeds is the assembly of a training population to exploit phenotypic variability and improve the prediction accuracy. Multi-breed predictions are more complex than single-breed predictions, and the use of a CV strategy that exploits the potential of different breeds improved the accuracy of genomic prediction [7,8]. We hypothesized that this approach could be potentially transposed at the phenotypic level to improve the accuracy of FTIR predictions. Therefore, the aim of this study was to investigate the effect of combining phenotypic information from different specialized and dual-purpose breeds in the training population to predict difficult-to-measure phenotypes not directly measured from milk (body condition score [BCS] and serum β-hydroxybutyrate [BHB]) and those directly measured in milk, (kappa casein—k-CN), from FTIR spectra to maximize the accuracy in the major cattle breed, i.e., Holstein.

## 2. Materials and Methods

### 2.1. Ethics Approval

This study did not require any specific ethics permit. The cows sampled belonged to private commercial herds and were not experimentally manipulated. Milk samples and blood samples were collected during routine milk recording carried out by technicians from the Breeders Federation of Trento Province (FPA, Trento, Italy), and were therefore authorized by a local authority.

### 2.2. Data

Phenotypic records were obtained from 1461 cows (specialized and dual-purpose breeds) belonging to 41 multi-breed dairy farms located in the province of Trentino (northeastern Italy) as part of a broader project (Cowplus project), described by Cecchinato et al. [11], investigating cattle farming in mountain areas. All the cows were enrolled in the milk-recording program of the Provincial Federation of Breeders (FPA, Trento, Italy) and monitored for milk production. The multi-breed dairy farms operate according to different production systems: small, traditional farms in the mountainous areas, and farms with larger, modern operations [12]. The 41 selected farms comprised from 1 to 5 breeds, which could be divided into two groups: (i) specialized dairy breeds—Holstein (31 herds, 468 cows) and Brown Swiss (35 herds, 657 cows); and (ii) dual-purpose breeds—Simmental (20 herds, 157 cows), Alpine Grey (14 herds, 75 cows), and Rendena (9 herds, 104 cows). Milk production was recorded by the official milk recording system.

Milk samples and phenotypic records were collected from one herd per day. Cow health status was determined based on rectal temperature, heart rate, respiratory profile, appetite, and fecal consistency. Only cows that were clinically healthy at the time of the visit were included in the study. Milk samples (50 mL) were collected from each cow during the evening milking and either (i) maintained at 4 °C (without preservative) until processing (within 24 h) at the Department of Agronomy, Food, Natural Resources, Animals, and Environment (DAFNAE) of the University of Padua, or (ii) stored at −80 °C until chromatographic analyses at DAFNAE’s Central Chemical Laboratory (LaCHI). To quantify BHB, blood samples were collected by a veterinarian via jugular venepuncture using vacutainer tubes. On the same day as milk sampling, BCS was assessed by technicians from the Breeders Federation of Trento Province (FPA, Trento, Italy). Milk FTIR spectra were stored during the milk sampling by the Breeders Federation of Trento Province (Trento, Italy).

Prediction in Holstein cows of three different phenotypes with different biological meanings and variabilities, i.e., BCS, serum BHB, and k-CN in % N, were assessed using an ensemble method (gradient boosting machine, GBM), with alternative cross-validation designs (within-breed, across-breed, and multi-breed).

Body condition score (BCS) was measured by a trained operator on the same day as milk sampling in cows, defined as days in milk (DIM), ranging from 10 to 380 days, based on Edmonson et al.’s [13] methodology, which classifies cows on a scale ranging from 1 (emaciated) to 5 (extremely fat).

β-Hydroxybutyrate (BHB, mmol/L) was measured in individual blood samples collected by jugular venepuncture using vacutainer tubes without anticoagulant. The blood samples were centrifuged at 1780× *g* for 10 min at 4 °C and stored at −20 °C until analysis at the laboratory of the Department of Animal Medicine, Production, and Health of the University of Padua (Padua, Italy). Blood BHB level was determined by the Ranbut RX Monza test (Randox, Crumlin, UK) on a Cobas C-501 analyzer (Roche Diagnostics, Mannheim, Germany).

Kappa casein (k-CN, % N) was measured in individual milk samples collected during the evening milking and stored without preservative at −80 °C until protein fraction analysis at DAFNAE, University of Padua. The separation of milk proteins was performed using validated reversed-phase high-performance liquid chromatography (RP-HPLC), as proposed by Maurmayr et al. [14], and expressed as a percentage of the total milk nitrogen content (% N).

Phenotypic quality control was performed for each breed separately by removing observations outside the interval between 3.0 standard deviations below and above the mean. After quality control, normal distribution for each phenotype was checked and at least five animals of each breed in a herd were required for inclusion in the analysis.

### 2.3. Infrared Milk Spectra

**Figure 1:**
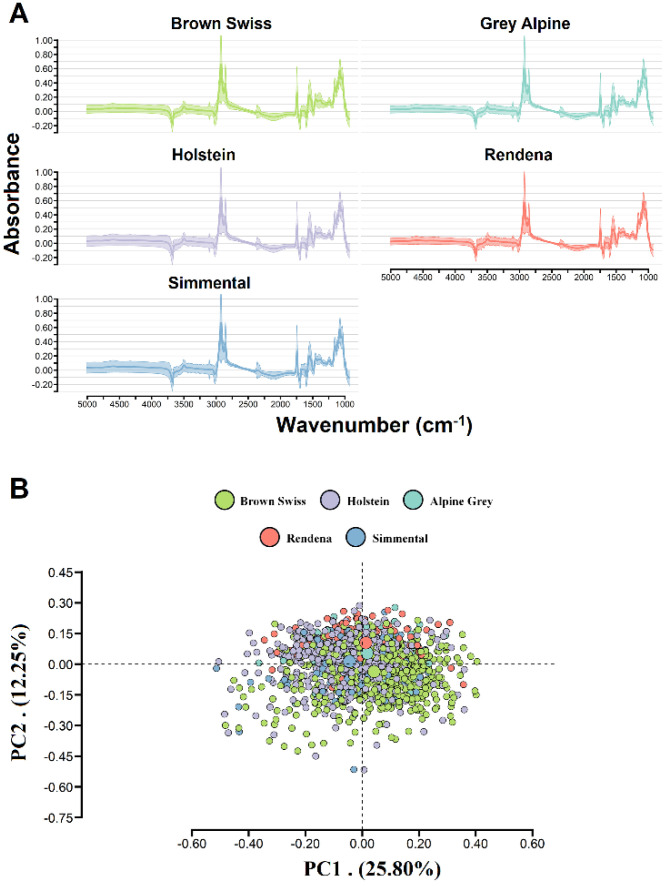
(**A**) Average value for Fourier-transform infrared (FTIR) spectra absorbance across specialized and dual-purpose breeds (solid line represents the average and color region represents the mean ± 3.5 × SD) and (**B**) principal component (PC) for the FTIR spectral data of milk samples recorded on specialized dairy breeds (Holstein and Brown Swiss) and dual-purpose breeds (Simmental, Alpine Grey, and Rendena). The big dot represents the PC means, and Holstein and Simmental breeds are overlapping the mean.

Spectra were obtained from the milk of all cows using a MilkoScan FT6000 (Foss A/S, Hillerød, Denmark) in the laboratory of the Breeders Federation of Trento Province (northeastern Italian Alps). Two milk spectra per animal were collected, covering the region from the short-wavelength infrared (SWIR) to the long-wavelength infrared (LWIR), with a total of 1060 spectral points in the region from 5011 to 925 cm^−1^ [15]. Milk spectra transmittance (T) was transformed to absorbance (A) using the equation A = log(1/T), and the two spectra from animals within each breed were then averaged before data analysis (Figure 1A). A principal component analysis integrating Mahalanobis distance was performed on the FTIR spectral data to remove possible outliers, at probability level < 0.01, according to Shah and Gemperline [16]. After quality control for phenotypic information and milk spectra through Mahalanobis distance, data from 460 Holstein, 646 Brown Swiss, 155 Simmental, 99 Rendena, and 73 Alpine Grey cows were included in the subsequent analyses (Figure 1).

### 2.4. Design of the Cross-Validation (CV) Populations

Prediction of the three target phenotypes in Holstein cows was assessed using within-breed (10-fold_HO), across-breed (BS_HO), and multi-breed (BS+HO_10-fold and Multi-breed) CV strategies.

Within-breed (10-fold_HO): The Holstein dataset was randomly split into 10 non-overlapping folds of approximately equal size. The training population consisted of 9 folds (414 cows for BCS, 407 for BHB, and 358 for k-CN), and the testing population was made up of the remaining fold (46 cows for BCS, 45 for BHB, and 35 for k-CN). To evaluate the reliability of the model, the cross-validation process was repeated ten times with each fold used as the testing population only once. We calculated predictive ability in each repetition, as well as the average across 100 estimates (10 folds and 10 replications) and the standard deviation.

Across-breed (BS_HO): Brown Swiss cows were assigned to the training dataset in order to develop the calibration equations for BCS (646 cows), BHB (620 cows), and k-CN (520 cows), and these equations were then used to predict the three target phenotypes in the whole Holstein population for BCS (460 cows), for BHB (468 cows), and for k-CN (393 cows). This CV scenario was used to evaluate the possibility of using a prediction equation from a specialized breed in Holstein cattle.

Breed combination: this CV design included three scenarios:

(i) BS+HO_10-fold, using only specialized dairy breeds (Brown Swiss and Holstein): the training population consisted of all the available Brown Swiss cows (646 cows for BCS, 620 cows for BHB, and 520 cows for k-CN) and 9 folds of the Holstein population (414 cows for BCS, 423 for BHB, and 358 for k-CN), while the testing population consisted of the remaining fold of the Holstein population (46 cows for BCS, 45 for BHB, and 35 for k-CN). We calculated predictive ability in each repetition, as well as the average across 100 predictions (10 folds and 10 replications), and the standard deviation.

(ii) Multi-breed, where both specialized and dual-purpose dairy breeds were assigned to the training population. In this scenario, Brown Swiss (646 cows for BCS, 620 cows for BHB, and 520 cows for k-CN), Simmental (154 cows for BCS, 155 cows for BHB, and 93 cows for k-CN), Rendena (99 cows for BCS, 97 cows for BHB, and 95 cows for k-CN), Alpine Grey (73 cows for BCS, 73 cows for BHB, and 68 cows for k-CN) and 9 folds of the Holstein cows (414 cows for BCS, 423 for BHB, and 358 for k-CN) comprised the training population, while the testing population was comprised of the remaining fold of the Holstein cows (46 cows for BCS, 45 for BHB, and 35 for k-CN). As previously described, each Holstein fold was used as the testing population only once, the entire analysis was repeated ten times, and the average predictive ability and its standard deviation were calculated from 100 replications.

(iii) Multi-breed CV2, in which both specialized and dual-purpose dairy breeds were assigned to the training population, and we fixed the number of animals in the training population to be similar to that of 10-fold CV (417 cows for BCS, 407 for BHB, and 358 for k-CN). To this aim, the training population from the Multi-breed CV was split into 3 folds, considering a total of 417 cows for BCS (Brown Swiss—189 cows, Holstein—116 cows, Simmental—51 cows, Alpine Grey—25 cows, and Rendena—33 cows), 423 for BHB (Brown Swiss—198 cows, Holstein—116 cows, Simmental—51 cows, Alpine Grey—25 cows, and Rendena—33 cows), and 358 for k-CN (Brown Swiss—167 cows, Holstein—103 cows, Simmental—33 cows, Alpine Grey—23 cows, and Rendena—32 cows). Each training fold was used to predict the phenotypes in the testing population as in the 10-fold HO CV (46 cows for BCS, 45 cows for BHB, and 35 cows for k-CN); this process was repeated 10 times for the testing population and three times for the training set. The average predictive ability and its standard deviation were calculated from 900 replications.

### 2.5. Statistical Method

Phenotypic prediction using FTIR spectra was assessed using GBM, a forward learning ensemble method that converts weak learners into strong learners by combining different predictors in a sequential way to reduce both bias and variance [17,18,19]. GBM sequentially builds regression trees with some shrinkage and variable selection, and each new model is added to the previous model with the aim of reducing the predictive error of the prior tree model considering dependencies among the trees [17,20,21]. The GBM model can be represented as follow:

$$\hat{y}=\overset{M}{\underset{m=1}{\sum }}\beta_{m}b\left(x,\gamma_{m}\right)$$

where $\hat{y}$ is the target phenotype (BCS, BHB, or k-CN); M is the number of iterations (expansion coefficients); $\beta_{m}$ are the function increments, also called “boosts”; and $b\left(x,\gamma_{m}\right)$ are the base learners, simple functions of the multivariate argument x with a set of parameters $\gamma_{m}=\left\{\gamma_{1},\;\gamma_{2},\ldots ,\gamma_{m}\right\}$. In GBM, expansions of the coefficients ${\left\{\beta_{m}\right\}}_{1}^{M}$ and parameters ${\left\{\gamma_{m}\right\}}_{1}^{M}$ are used to map associations between FTIR predictor variables (x) and the target phenotype (y), considering the joint distribution of all values (y,x) that minimise the loss function $L\left\{y_{i},F\left(x\right)\right\}$, given $\left[y,F_{m-1}\left(x_{i}\right)+h\left(y_{i};x_{i},p_{m}\right)\right]$, where $p_{m}$ is the FTIR (only 1 FTIR spectrum is selected at each iteration) that minimises the $\overset{n}{\underset{i=1}{\sum }}L\left[y,F_{m-1}\left(x_{i}\right)+h\left(y_{i};x_{i},p_{m}\right)\right]$. GBM uses the algorithm specified by Hastie et al. [17]. GBM analyses were performed using the h2o R package (https://cran.r-project.org/web/packages/h2o, accessed on 7 May 2021).

**Figure 2:**
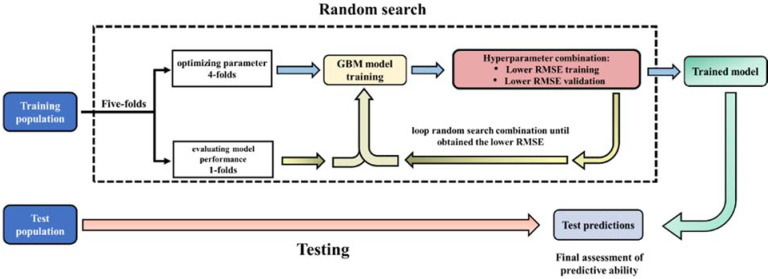
An illustration of a random search of hyperparameter optimization for a gradient boosting machine (GBM). The general process includes splitting the training population from each validation scenario (10-fold_HO, BS_HO, BS+HO_10-fold, Multi-breed, and Multi-breed CV2) into 5 folds, aiming to optimize the main parameters of the GBM approach to accurately learn the mapping from the milk spectra data to the target trait, i.e., lowest root mean square error estimation (RMSE). Thus, the trained model is then evaluated on the disjointed test population from each cross-validation scenario.

The predictive performance of the GBM method depends on four parameters to minimize the error of predictions on the validation subset. These parameters are: (1) the number of trees (*Ntree* represents the total number of trees in the sequence used in the model), (2) learning rate (determines the contribution of each tree to the final model and performs shrinkage to avoid variable overfitting), (3) maximum tree depth (establishes the level of complex interactions between predictors), and (4) minimum samples considered in each leaf (controls the complexity of each tree). The *Ntree* values used in the random search ranged from 10 to 8000 in intervals of 10, the learn rate ranged from 0.001 to 1 in intervals of 0.001, maximum tree depth was determined using the values from 1 to 80 in intervals of 1, minimum samples per leaf was determined from 1 to 100 in intervals of 5. We performed a random grid search of hyperparameters using the h2o.grid function in the h2o R package (https://cran.r-project.org/web/packages/h2o, accessed on 7 May 2021) in order to select the optimal hyperparameters combination that minimizes the predictive loss function (i.e., prediction error) of the model for each trait (Figure 2). The random grid search was performed using the training set from each CV design (10-fold_HO, BS_HO, BS+HO_10-fold, Multi-breed and Multi-breed CV2) for each trait, splitting it into a 5-fold CV [22]. Thus, 4 folds were assigned to hyperparameter optimization, aiming to find the best combination of the main hyperparameters for GBM approach, while the 1 remaining fold was used to evaluate the model performance based on the loss function (root mean square error—RMSE) and prediction accuracy (r-square—r^2^) [19]. After finding the best-trained model with the lowest root mean square error (RMSE) and highest prediction accuracy (r^2^), it was applied to a disjointed testing population for each CV scenario (10-fold_HO, BS_HO, BS+HO_10-fold, Multi-breed, and Multi-breed-CV, previously described in Section 2.4 Design of the Cross-Validation) to obtain the final prediction parameters (Figure 2).

### 2.6. Assessment of Model Performance

The predictive ability of the GBM across the CV scenarios was assessed by Pearson’s correlation ($r_{p}$) between the observed and predicted phenotypes, root mean square error (RMSE), and the bias of prediction on the testing dataset. To assess the differences in predictive ability ($r_{p}$) across CV scenarios, evaluation was performed using a Hotelling–Williams *t*-test [23]. The unbiasedness of the prediction was given by the slope of the linear regression of the observed and predicted values in each cross-validation design. The mean percentage error (MPE%) was used as another model bias parameter: $(MPE\left(\%\right)=\overset{n}{\underset{i=1}{\sum }}\left(\frac{{\bar{y}}_{i,obs}-y_{i,pred}}{y_{i,obs}}\right)\times \frac{100}{n}$), where ${\bar{y}}_{i,obs}$ is the average value of the observed phenotypic information, $y_{i,pred}$ is the predicted value in the testing population, and n is the number of animals with phenotypes predicted in the testing population. We also evaluated the relative difference (RD) in predictive ability, calculated as $RD=\frac{\left(r_{m}-r_{C}\right)}{r_{C}}\times 100$, where $r_{m}$ represents model performance using the BS_HO, BS+HO_10-fold, and Multi-breed CV scenarios, and $r_{C}$ represents model performance using 10-fold_HO CV for the training scenario. Fisher’s Z-transform test based on Zou [24] was used to determine the significance level of the differences in predictive ability (Pearson’s correlation) between the BS_HO, BS+HO_10-fold, Multi-breed, and Multi-breed CV2 CV scenarios and the 10-fold_HO.

## 3. Results

### 3.1. Phenotypic and FTIR Spectra Information

The across-breed descriptive statistics for BCS, BHB, and k-CN are shown in Table 1. Specialized breeds (Holstein and Brown Swiss) had lower BCS than dual-purpose breeds (Simmental, Rendena, and Alpine Grey), with the Holstein breed differing significantly from the dual-purpose breeds (*p* < 0.0005) and the Brown Swiss differing significantly from the Simmental (*p* < 0.0035). Simmental cows had the highest value for serum BHB (0.62), statistically different from the other breeds (*p* < 0.005), while the Brown Swiss and Alpine Greys had the highest values for k-CN proportions (16.13% N and 15.34% N, respectively), which were also statistically different from the other breeds (*p* < 0.01), and Holstein cows had the lowest phenotypic values (13.73% N; Table 1). Principal component analysis (PCA) was applied to the milk FTIR spectra to visualize the differences across breeds (Figure 1B). The first two principal components (PCs) accounted for 25.80% and 12.25% of the FTIR spectra variability, respectively. We observed no differences in FTIR spectra among breeds, indicating similarity across the specialized and dual-purpose breeds (Figure 1B). Comparing the mean values for the principal components (i.e., the big dots in Figure 1B), we observed a greater similarity between the Simmental and the Holstein breeds against the other breeds. However, the distance existing between Holstein and Brown Swiss was comparable to the distance observed between Holstein and dual-purpose breeds, except for Simmental breed.

### 3.2. Cross-Validation Scenarios

**Figure 3:**
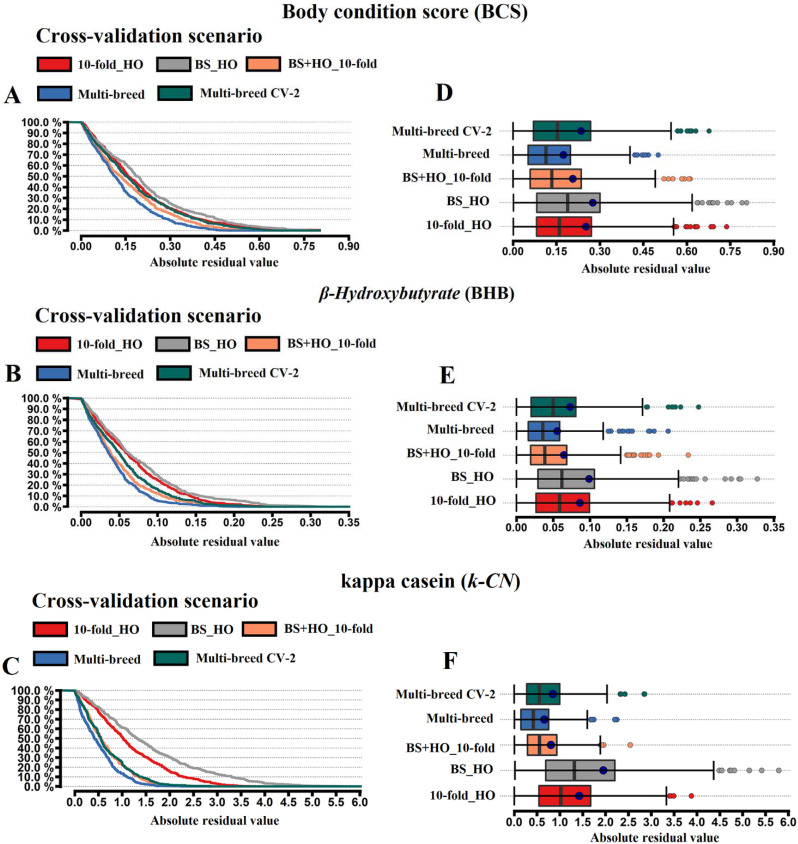
Residual diagnostics comparison across cross-validation designs for the FTIR prediction of body condition score (BCS), blood *β-Hydroxybutyrate* (BHB, mmol/L), and kappa casein (k-CN, % N). Absolute residual distribution ((**A**) for BCS, (**B**) for BHB and (**C**) for k-CN) and box plot of the absolute errors ((**D**) for BCS, (**E**) for BHB and (**F**) for k-CN) between the observed and predicted phenotypic values.

The within-breed (10-fold_HO), across-breed (BS_HO), and multi-breed (BS+HO_10-fold, Multi-breed, and Multi-breed CV2) cross-validations were compared on the basis of the model fit parameters (Table 2). The sizes of both the training and validation sets of each CV scenario are reported in Supplementary Figure S1. The accuracies of the FTIR predictions for BCS, BHB, and k-CN using the different CV strategies are shown in Table 2. Prediction accuracies obtained with BS_HO were 7.5% lower than 10-fold_HO (Table 2 and Figure 3). Interestingly, the use of multi-breed training sets increased phenotypic prediction accuracy by around 6.5% (BS+HO_10-fold) and 8.5% (Multi-breed) (Table 2). To demonstrate the effect of the CV scenarios on predictive ability, the relative difference (RD) was assessed comparing the alternatives CV against the 10-fold Holstein. Including different breeds in the training set led to an increase in model predictive performance, which was more evident with the multi-breed scenario (8% for BCS, 7% for BHB, and 10% for k-CN). On the other hand, splitting the multi-breed training population to consider an equal number of animals as in the 10-fold_HO training population led to a slight relative difference among the 10-fold_HO and Multi-breed CV2 scenarios (−1.23% for BHB, −0.47% for BCS, and 2.47% for k-CN; Table 2).

When we investigated the significance of these relative differences using the Hotelling–Williams *t*-test, we found the RDs were significant for all traits (*p* < 0.05), except for BCS with the BS+HO_10-fold CV, although here the increase was 4.76% with Pearson’s correlation (Table 2). The relative gains observed with the multi-breed scenario were significantly higher than with all the other CV scenarios (*p* < 0.005).

### 3.3. Bias and Predictive Error Parameters of the Cross-Validation Scenarios

The coefficient of regression (slope) of the observed values on the predicted values was calculated as a measure of the bias of each CV scenario. A value of bias equal to one is ideal, indicating unbiased predictions [25]. For all traits, the slopes of all the CV scenarios were not significantly different from one, indicating no significant bias in the predictions (Table 2). Nonetheless, the slope value of the BS_HO CV scenario was slightly higher than 1.1 for BCS and k-CN traits (average across traits 1.18), and lower than 0.95 for BHB. Notably, with the larger training populations, i.e., the Multi-breed CV scenario, there were more unbiased predictions (i.e., closer to 1) than with the other cross-validation designs.

Predictive error parameters (MPE and RMSE) showed that FTIR predictions with the BS+HO_10-fold and multi-breed scenarios led to lower residual parameters (Table 2). Model fit assessment using RMSE indicated that the multi-breed CV considerably reduced the predictive error, from 8% to 22% for Multi-breed and from 0.1% to 11% for BS+HO_10-fold. Both the BS+HO_10-fold and Multi-breed CV scenarios had higher probabilities of lower residual values compared with 10-fold_HO and BS_HO scenarios (Figure 3). The use of a predictive equation developed in a specific breed, i.e., the BS_HO CV, resulted in a higher number of extreme residual values, suggesting more biased predictions (Figure 3). Using the BS_HO CV scenario, we observed the highest MPE estimation, mainly for k-CN trait with −21.09%, indicating that this scenario CV led to a higher overestimation of the prediction, leading to a slope of 1.21 (Table 2).

## 4. Discussion

Assembling sufficiently large training populations to make accurate FTIR predictions is a major challenge for high-throughput phenotyping in dairy cattle, especially for traits that are difficult or time-consuming to measure. To overcome this problem, we investigated the feasibility of using combinations of different populations and/or different breeds in the training set to obtain greater variability in the available information, and hence improve the prediction accuracy of FTIR spectra. Specifically, we evaluated the performance of within-breed, across-breed, and multi-breed FTIR training sets for phenotypic prediction in the Holstein breed. The results show that combining different breeds in the training set greatly increased FTIR prediction accuracy. Moreover, the phenotypic variability and composition of the training population had a large impact on prediction performance.

Using a pure Brown Swiss training population to predict phenotypes in Holstein resulted in lower accuracies compared with using Holsteins in both the training and validation sets. Although, the predictive equation that was developed exhibited poor predictive ability, there were no differences in FTIR variability between these two specialized dairy breeds. This means that the major factor affecting model predictive performance is probably related to phenotypic differences between training and validation individuals (Supplementary Figure S2). These observed differences in phenotypes across breeds were an important factor in the reductions in predictive ability of 10% for BCS, followed by 6.19% for BHB and 6.18% for k-CN when a specific breed was used as the training set (BS_HO scenario) for predictions in the Holstein breed (Table 2). Attaining high predictive ability (*r_p_* > 0.55) for FTIR prediction is of interest in the context of genetic selection for the target traits in dairy breeding programs due to the fact that its high accuracy is associated with the highest genetic relationship between measured and predicted traits [26]. The predictive accuracy observed was affected by differences in the number of observations and the extent of phenotypic variability in the training population used to develop the calibration equation (Table 2). In this framework, McParland et al. [27] and Maurice-Van Eijndhoven [28] observed that use of the calibration equation was similarly limited in phenotype and spectra variability among the training and validation subsets. The authors reported that inclusion of different Holstein populations [27] and different breeds [28] in the training population used to create the calibration equations for phenotype prediction is of paramount importance in obtaining more accurate predictions. Differences in milk composition across different breeds is an additional important factor, as the FTIR wavelengths extract interpretable information linking the complex presence of specific chemical bonds in milk and the target phenotype. In this case, the calibration equations were less accurate in linking either the complex trait or the spectral data from the training data with the validation data set.

The BS_HO design, where the training population was Brown Swiss and the validation population Holstein, was not useful as it led to poor FTIR predictions, caused by the phenotypic differences between the breeds (Table 2). Studies comparing different CV strategies using a single breed, where the training and validation sets overlapped to a greater (k-folds) or lesser (leave-one-herd-out) extent, confirmed the hypothesis that where there is less overlap prediction accuracy decreases, the residual parameters increase (MPE and RMSE), and there are more biased predictions [4,29]. In principle, FTIR prediction performance could be affected by CV strategies and the inherent nature of the target phenotype, as well as by the populations assigned to the training and validation sets [17]. Major factors accounting for the reduction in predictive ability with the BS_HO CV design could be the number of animals in the training and testing populations (Supplementary Figure S1) and phenotypic differences between the Brown Swiss (training set) and Holstein cows (testing population; Table 1).

The prediction ability of FTIR is directly associated with the number of animals in the training population (Supplementary Figure S1) and similarity in terms of phenotypic variation between the training and validation populations. Here, when the equations were calibrated using the target Holstein cows (9 folds) jointly with the Brown Swiss cows (BS+HO_10-fold) or using Brown Swiss plus dual-purpose dairy breeds (Multi-breed), with the aim of increasing the training population, the predictions were more accurate and robust, which shows that better performance is obtained by combining breeds in the training set than by using single (10-fold_HO) or specific breeds (BS_HO). However, when the CV scenario was designed to combine the specialized and dual-purpose breed, maintaining a similar number of animals in the training population as 10-fold_HO, a slight difference was observed, which evidenced that not only the phenotypic variability but also the size of the training set is essential to build robust predictions (Table 2). This could be explained by the greater number of animals in the training population and by the closely related phenotypic values in the training and testing populations, which could be a key factor in increasing predictive ability and obtaining unbiased FTIR predictions [2]. It seems, therefore, that differences in FTIR prediction accuracies are linked to differences in phenotypic and spectral variability [28]. Increasing the number of training cows and the phenotypic variability seems to be important for more accurately determining the contribution of FITR to phenotypic variability and also for distinguishing their effects from random noise regions. Compared with the 10-fold_HO, FTIR predictive ability with the BS+HO_10-fold and Multi-breed CV scenarios increased by 6% and 9%, respectively.

This increase in prediction accuracy when using an admixture of breeds in the training population could be a useful tool to predict phenotypes with economic importance in dairy cattle. Our results show that training populations consisting of different breeds, including the target breed (Holstein), represent an efficient way of increasing the accuracies of predictions (Table 2). For multi-breed predictions, it is crucial that milk spectra and phenotypic information exhibit the same variability.

The design of the training population using different breeds had a strong influence on the prediction accuracy (Figure 1B). It was observed that using a single specialized dairy breed (i.e., Brown Swiss) BS_HO provided lower predictive ability against 10-fold_HO, which was confirmed by the difference in PCA average (Figure 1B). The observed differences between Holstein and Brown Swiss breeds may be due to differences in milk composition, which are captured by FTIR spectroscopy. This led to a general decrease in accuracy by around 10% for BCS and 6% for BHB and k-CN, and an increase in prediction error by 18% for RMSE with an overestimation of around 168% for BCS and k-CN and underestimation of 139% for BHB based on the MPE parameter (Table 2), which could be due to the FTIR relationship between breeds (Figure 1B). We have also shown that increasing the number of animals in the training population (BS+HO10-folds and Multi-breed) increased prediction accuracy and reduced prediction errors (MPE and RMSE), leading to a less unbiased prediction (MPE) with a slope slightly different from 1 (Table 2). On the other hand, the Multi-Breed CV2, which included different breeds in the training set, keeping the same number of animals as the 10-folds_HO scenario, did not improve accuracy, indicating that the variability and size of the training set represent the main factors to improve the prediction accuracy. Overall, the effectiveness of FTIR predictions in small training populations can be improved by increasing the variability and size of the training set and target population. These results suggest that FTIR spectra (when prediction accuracy is moderate to high) may represent a valid alternative to “standard” phenotyping and can be exploited in dairy breeding programs for traits that are expensive and difficult to measure, achieving a similar or slightly inferior genetic response to the measured traits [30,31]. Furthermore, the use of multi-breed CV scenarios seemed to improve prediction accuracy, explaining a greater proportion of the phenotypic variation of the target trait.

## 5. Conclusions

Our findings confirm that accurate Fourier-transform infrared-based predictions in dairy cattle can be achieved by increasing the size and the phenotypic variability of the training population. Our comparison of different validation strategies showed that phenotypic prediction of Holstein records using a pure Brown Swiss training set resulted in the worst performance, while the best performance was obtained with Multi-breed training populations that included Holstein animals, which increased predictive ability by 6% to 8%. Overall, these results indicate that validation scenarios using combinations of different dairy breeds constitute a promising strategy to improve Fourier-transform infrared-based phenotypic prediction. Moreover, they open the possibility of using a similar approach for improving the phenotypic prediction accuracy also in minor cattle breeds.

## Figures and Tables

**Table 1:** Descriptive statistics for body condition score (BCS), blood β-hydroxybutyrate (BHB, mmol/L), and kappa casein (k-CN, % N) assessed in specialized and dual-purpose breeds.

Breed	N	Mean ^1^	SD	Min	Max
BCS					
Holstein	460	2.81 ^c^	0.324	2.00	3.75
Brown Swiss	646	2.96 ^b^	0.339	2.00	4.00
Simmental	154	3.06 ^a^	0.342	2.50	4.00
Rendena	99	2.97 ^ab^	0.346	2.00	3.75
Alpine Grey	73	3.07 ^ab^	0.342	2.50	4.00
β-hydroxybutyrate (BHB, mmol/L)					
Holstein	449	0.55 ^bc^	0.163	0.22	1.01
Brown Swiss	620	0.58 ^b^	0.136	0.32	1.03
Simmental	155	0.62 ^a^	0.146	0.34	1.01
Rendena	97	0.53 ^c^	0.112	0.32	0.90
Alpine Grey	73	0.56 ^bc^	0.109	0.33	0.87
Kappa casein (k-CN, % N)					
Holstein	392	13.73 ^c^	2.151	8.27	20.15
Brown Swiss	520	16.13 ^a^	1.632	11.31	21.40
Simmental	93	14.25 ^b^	1.386	9.83	17.80
Rendena	95	14.53 ^b^	2.219	8.89	19.55
Alpine Grey	68	15.34 ^b^	1.882	10.60	20.18

N—number of samples, SD—standard deviation, Min—minimum, Max—maximum. ^1^ Different letters represent significant differences (*p* < 0.05, Tukey test).

**Table 2:** Average model predictive performance and its standard deviation values (in parentheses) ^1^ obtained using gradient boosting machine (GBM) for phenotypic prediction of body condition score (BCS), blood β-hydroxybutyrate (BHB, mmol/L), and kappa casein (k-CN, % N), with the prediction of Holstein Friesian as the final target.

Trait	Model Fit ^1^	Validation Strategies for Holstein Prediction ^2^				
		10-fold_HO	BS_HO	BS+HO_10-fold	Multi-Breed	Multi-Breed CV2
BCS	$r_{p}$	0.63 (0.023) ^b^	0.57 ^c^	0.66 (0.025) ^ab^	0.68 (0.022) ^a^	0.63 (0.036) ^b^
	RD (%)	−	−9.52	4.76 (0.025)	7.94 (0.022)	−0.47 (0.036)
	MPE	−1.54 (0.882)	−3.33	0.84 (0.869)	0.70 (0.664)	−1.05 (0.904)
	RMSE	0.25 (0.017)	0.28	0.25 (0.019)	0.23 (0.015)	0.27 (0.031)
	slope	1.07 (0.030)	1.15	0.97 (0.029)	0.99 (0.023)	1.10 (0.093)
BHB	$r_{p}$	0.80 (0.023) ^bc^	0.75 ^c^	0.85 (0.027) ^ab^	0.87 (0.025) ^a^	0.79 (0.035) ^bc^
	RD (%)	−	−7.41	4.94 (0.027)	7.41 (0.025)	−1.23 (0.035)
	MPE	−0.96 (2.085)	2.29	−0.62 (2.033)	−0.39 (0.851)	0.89 (1.046)
	RMSE	0.09 (0.009)	0.10	0.08 (0.009)	0.07 (0.006)	0.09 (0.010)
	slope	1.03 (0.026)	0.94	1.05 (0.028)	1.00 (0.021)	0.97 (0.033)
k-CN	$r_{p}$	0.81 (0.025) ^b^	0.76 ^c^	0.87 (0.022) ^ab^	0.88 (0.023) ^a^	0.82 (0.076) ^b^
	RD (%)	−	−11.25	8.75 (0.022)	9.99 (0.023)	2.47 (0.076)
	MPE	−6.82 (3.356)	−21.09	−5.33 (3.061)	−2.94 (1.983)	3.56 (2.851)
	RMSE	1.08 (0.052)	1.42	0.96 (0.036)	0.84 (0.027)	1.01 (0.107)
	slope	1.06 (0.034)	1.21	1.08 (0.039)	1.00 (0.029)	0.95 (0.059)

^1^$r_{p}$ —Pearson’s correlation; RD—represents the relative difference in predictive ability of CV scenarios against the 10-fold_HO in percentage; MPE—mean percentage error; RMSE—root mean square error; slope—represents the slope value of regression of observed and predicted value. Different letters represent the statistical difference based on Hotelling–Williams *t*-test (*p* < 0.05). ^2^ 10-fold_HO—Holstein cattle were split in 10 folds of approximately equal size, where 9 folds were used to generate the prediction equations and tested on 1 fold of Holstein, and the average model predictive performance and its standard deviation were obtained from 100 replications; BS_HO—Brown Swiss breed was used as a training set to create the prediction equations; BS+HO10-fold—Brown Swiss cattle and 9 folds of Holstein cross-validation was used to generate the prediction equations and tested on 1 fold of Holstein, and the average model predictive performance and its standard deviation were obtained from 100 replications; and Multi-breed—specialized dairy breeds (9 folds of Holstein and Brown Swiss) and dual-purpose breeds (Simmental, Alpine Grey, and Rendena) were used as the training set to predict 1 fold of Holstein cattle and the average predictive ability and its standard deviation were calculated from 100 replications. Multi-breed CV2—specialized dairy breeds (9 folds of Holstein and Brown Swiss) and dual-purpose breeds (Simmental, Alpine Grey, and Rendena) were split into 3 folds and then used as the training set to predict 1 fold of Holstein cattle and the average predictive ability and its standard deviation were calculated from 900 replications.

## Data Availability

The phenotypic and FTIR spectral data are available for academic use from the authors upon reasonable request.

## Supplementary Materials

The following are available online at https://www.mdpi.com/article/10.3390/ani11071993/s1, Supplementary Figure S1. Number of animals considered as training population and validation set for body condition score (BCS), β-hydroxybutyrate (BHB, mmol/L), and kappa casein (k-CN, % N) using different CV schemes: 10-folds, BS_Holst, BS+HO10-fold, Multi-breed, and Multi-breed CV2. Supplementary Figure S2. Boxplot and histogram of the phenotypic values for body condition score (BCS—A), blood β-hydroxybutyrate (BHB—B), and κ-casein expressed as % N (k-CN—C), respectively, for across-breed cross-validation design (BS_Holst), in which the Brown Swiss cows were assigned as the training set and Holstein as the validation set.
