# Supplementary material for: Comparison of Single-Breed and Multi-Breed Training Populations for Infrared Predictions of Novel Phenotypes in Holstein Cows
Source: Animals (Basel). 2021 Jul 2;11(7):1993. doi: 10.3390/ani11071993 (PMC8300349; doi:10.3390/ani11071993)
Supplement: Supplementary file 1 [file animals-11-01993-s001.zip › animals-1272957-supplementary.pdf]

Supplementary information

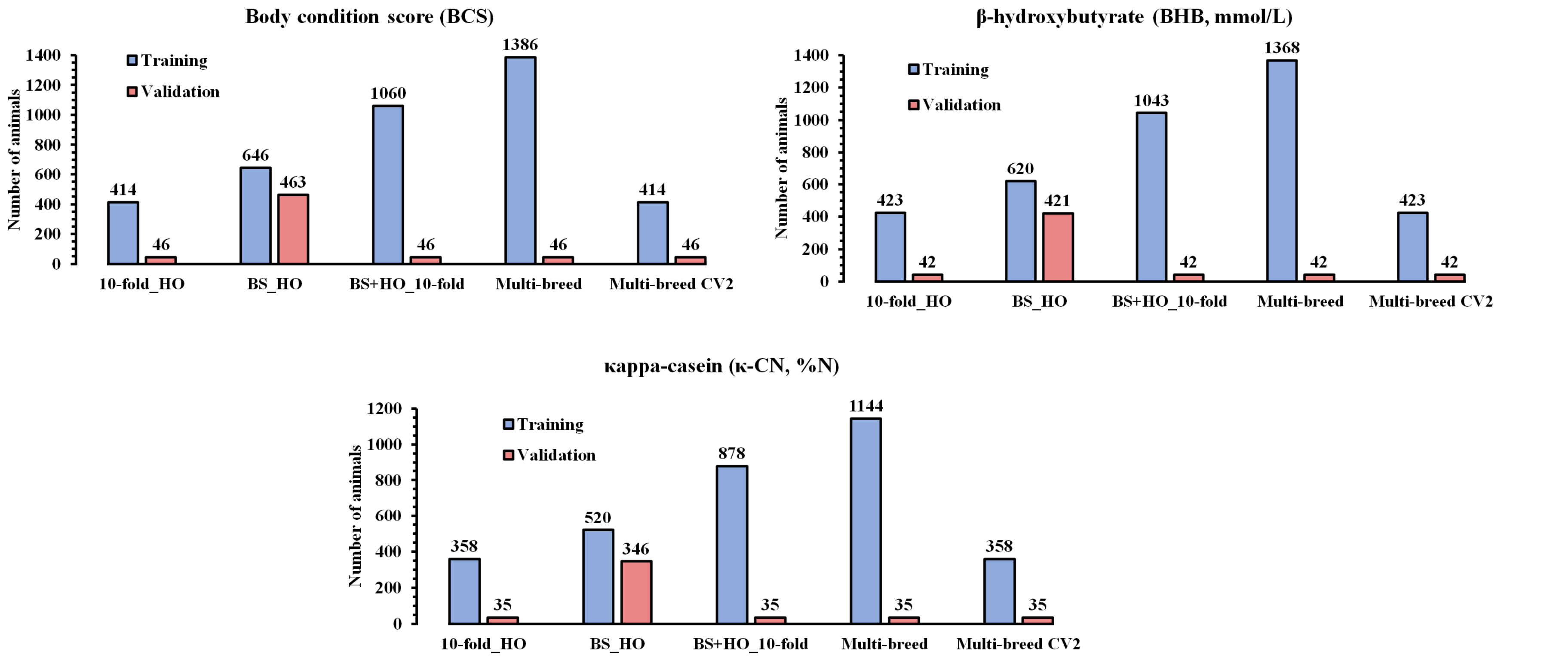

Supplementary Figure S1. Number of animals considered as training population and validation set for Body condition score (BCS), β-hydroxybutyrate (BHB, mmol/L), and kappa casein (*k*-CN, %N) using different CV schemes: 10-fold_HO, BS_HO, BS+HO_10-fold, Multi-breed and Multi-breed CV2.

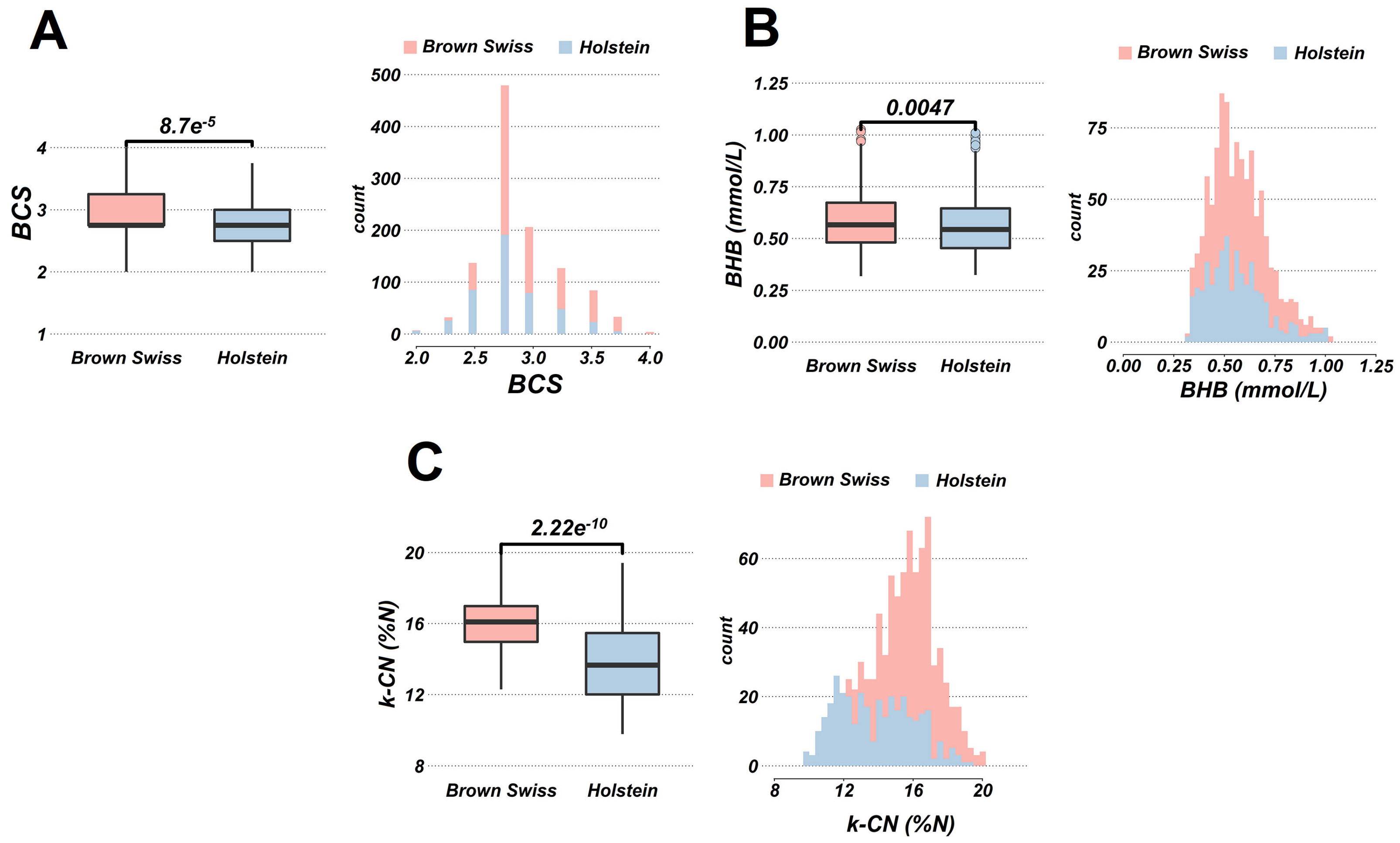

Supplementary Figure S2. Boxplot and histogram of the phenotypic values for body condition score (BCS – A), blood β-hydroxybutyrate (BHB – B), and κ-casein expressed as %N (κ-CN – C), respectively for across-breed cross-validation design (BS_Holst), in which the Brown-Swiss was assigned as training set and Holstein as validation set.
